# Caveolin Scaffolding Domain (CSD) Peptide LTI-2355 Modulates the Phagocytic and Synthetic Activity of Lung-Derived Myeloid Cells in Idiopathic Pulmonary Fibrosis (IPF) and Post-Acute Sequelae of COVID Fibrosis (PASC-F)

**DOI:** 10.3390/biomedicines13040796

**Published:** 2025-03-26

**Authors:** Brecht Creyns, BreAnne MacKenzie, Yago Amigo Pinho Jannini Sa, Ana Lucia Coelho, Dale Christensen, Tanyalak Parimon, Brian Windsor, Cory M. Hogaboam

**Affiliations:** 1 Division of Pulmonary & Critical Care Medicine, Department of Medicine, Women’s Guild Lung Institute, Cedars-Sinai Medical Center, Los Angeles, CA 90048, USA; brechtcreyns@gmail.com (B.C.); amigopinhy1@cshs.org (Y.A.P.J.S.); analucia.coelho@cshs.org (A.L.C.); tanyalak.parimon@cshs.org (T.P.); 2 Rein Therapeutics, Inc., 12407 N. Mopac Expy., Suite 250 #390, Austin, TX 78758, USA; breannemackenzie@rein.com (B.M.); bwindsor@reintx.com (B.W.); 3 Division of Hematology, Department of Medicine, Duke University, Durham, NC 27708, USA; dale.christensen@duke.edu

**Keywords:** IPF, macrophages, peptides, PASC-F, caveolin-1

## Abstract

**Rationale**: The role of the innate immune system in idiopathic pulmonary fibrosis (IPF) remains poorly understood. However, a functional myeloid compartment is required to remove dying cells and cellular debris, as well as to mediate innate immune responses against pathogens. Aberrant macrophage activity has been described in patients with post-acute sequelae of COVID fibrosis (PASC-F), and caveolin scaffolding domain (CSD) peptides have been found to attenuate inflammation and fibrosis in mouse lung injury models. Therefore, we examined, for the first time, the effects of CSD peptide LTI-2355 on the functional and synthetic properties of human myeloid cells isolated from lung explant tissue of donor lungs as well as IPF and PASC-F lung explant tissue. **Methods and Results**: CD45^+^ myeloid cells isolated from lung explant tissue from IPF and PASC-F patients exhibited an impaired capacity to clear autologous dead cells and cellular debris. The uptake of pathogen-coated bioparticles was impaired in myeloid cells from both fibrotic patient groups independent of the type of pathogen, highlighting an intrinsic functional cell impairment. LTI-2355 improved the phagocytic activity of both IPF and PASC-F myeloid cells, and this improvement was paired with decreased proinflammatory and pro-fibrotic synthetic activity. LTI-2355 was also shown to primarily target CD206-expressing IPF and PASC-F myeloid cells. **Conclusions**: Primary myeloid cells from IPF and PASC-F patients exhibit dysfunctional phagocytic and synthetic properties that are modulated by LTI-2355. LTI-2355 treatment of IPF myeloid cells resulted in significantly reduced sCD163, IFN-α2, IFN-γ, IL-2, IL-10, IL-12p40, and MMP-1 in the cell supernatant. This study highlights an additional mechanism of action of the CSD peptide in the treatment of IPF and progressive fibrotic lung disease.

## 1. Introduction

IPF is an aging-associated progressive fibrotic lung disease that is characterized by aberrant fibroblast and epithelial cell activity. The role of the immune system in the initiation and maintenance of this disease remains poorly understood. Myeloid cells, including macrophages and dendritic cells, are major innate immune cells that play a central role in lung homeostasis, the response to infection or injury, and tissue repair [1]. Lung-resident alveolar macrophages (TR-AMs) are the main AMs in this location, whereas in IPF, ontologically distinct AMs derived from circulating monocytes (Mo-AMs) drive the disease [2]. More recently, single-cell RNA sequencing (scRNAseq) efforts have identified activated myeloid cell subpopulations that contribute to the fibrotic response [3,4,5]. These scRNAseq analyses revealed at least four subpopulations based on unique transcript expression, including osteopontin (SPP1)^+^ and chitinase 3-like-1 (CHI3L1) high (^hi^) AMs that are specific to IPF lungs. Increased levels of SPP1^hi^ MER proto-oncogene tyrosine kinase (MERTK)^hi^ AMs were reported by [4], who also identified fatty acid-binding protein 4 (FABP4)^hi^ and FCN1^hi^ macrophages. Interestingly, SPP1^hi^ macrophages in IPF and chronic obstructive pulmonary disease (COPD) appear to have intermediate features between AMs and interstitial macrophages [3,6,7]. Several dendritic cell types were also observed in these scRNAseq studies. Overall, these published findings highlight the diverse nature of myeloid cell subtypes in IPF.

During the recent COVID-19 pandemic, infiltrating monocytes, macrophages, and other myeloid cells were identified as key inflammatory cells in patients suffering from severe disease [8,9,10]. In progressive COVID-19 (also known as long COVID-19) and post-acute sequelae of COVID (PASC), pro-fibrotic macrophages were identified with a gene signature that mirrored that of the pro-fibrotic macrophages identified in IPF [11]. Bosteels et al. [12] confirmed that there is a deficit of alveolar macrophages in PASC-F lungs, perhaps due to defective GM-CSF instruction. At present, the function and contribution of these myeloid cells to the fibrotic process in COVID-19 remain unclear.

Caveolin-1 (Cav-1) is a 20 kDa protein; it is a key protein in the formation of plasma membrane invaginations together with cavin proteins. These plasma membrane invaginations, or caveolae, are present in many cell types in the lung. A reduction in the abundance of caveolae has been shown to contribute to lung diseases, including asthma, fibrosis, COPD, acute lung injury, and inflammation [13]. The Cav-1 protein sequence consists of four domains: (a) an NH2-terminal domain, (b) a CSD (residues 82–101) carrying a cholesterol recognition/interaction consensus sequence; (c) a membrane domain, which interacts with membrane lipids; and (d) a COOH-terminal domain [14]. Cav-1 contributes to cell signaling pathways in healthy lungs. Decreased expression of Cav-1 is observed in both IPF and PASC-F lung tissue sections; this loss of Cav-1 leads to increased fibroblast activation [15,16]. In addition, *Cav-1* gene therapy has been shown to attenuate bleomycin-induced pulmonary fibrosis and reduce the infiltration of neutrophils and monocytes/macrophages [17]. Thus, Cav-1 has an important regulatory role in fibrosis in the lung.

To explore the role of Cav-1 in myeloid cell biology, we examined the effects of LTI-2355 on the functional and synthetic properties of IPF and PASC-F myeloid cells isolated from lung explants. LTI-2355 is a soluble and proteolysis-resistant 13-mer caveolin scaffolding domain (CSD) peptide with anti-fibrotic properties that has been used in several preclinical models. Specifically, we addressed the effect of LTI-2355 on both the functional and synthetic properties of adherent CD45^+^ myeloid cells enriched from healthy donors and lung explants from IPF and PASC-F patients.

## 2. Methods and Materials

CD45^+^ myeloid cell enrichment from healthy donors and explanted human lung samples. A single-cell solution was obtained from healthy human donors and IPF lungs after enzymatic digestion with 10× Liberase (Sigma, St. Louis, MO, USA, 5401127001) and DNase I (9003-98-9, STEMCELL Technologies, Vancouver, Canada) for 40 min at 37 °C in complete HBSS (21-023-CV, Corning; Corning, NY, USA). The enzymatic digestion was stopped with cold HBSS with bovine serum albumin (BSA), and single-cell preparations were obtained by passing the cell mixture through a PluriStrainer (500–70 µM cell strainers; 43-50500, PluriSelect, Leipzig, Germany) before blocking with Fc block for 10 min (422302, BioLegend, San Diego, CA, USA). CD45^+^ cells were enriched using anti-CD45 magnetic MicroBeads and LS columns (130-045-801 and 130-042-401, Miltenyi Biotec, Bergisch Gladbach, Germany). For CD206-positive (CD206^+^) cell enrichment, cells were incubated for 25 min with biotinylated anti-human MMR (321118, BioLegend, San Diego, CA, USA), washed, and conjugated with streptavidin microbeads (130-048-102, Miltenyi Biotec, Bergisch Gladbach, Germany) for 15 min. An institutional review board (IRB) at Cedars-Sinai Medical Center approved the use of de-identified human lung samples to enrich the CD45^+^ myeloid cells studied in the experiments described herein. The healthy donors and fibrotic patient characteristics are summarized in Table 1.

CD45^+^ myeloid cell enrichment and in vitro cultures. Myeloid cells enriched from healthy donors and IPF patient explant lung samples were allowed to adhere for 24 h in Dulbecco’s modified Eagle’s medium (DMEM, 12-604Q Lonza, Basel, Switzerland) supplemented with 10% FBS, 1% penicillin–streptomycin–amphotericin B (17-754E, Lonza, Basel, Switzerland), 0.2% Primocin (Invivogen, San Diego, CA, USA), and 200 mM L-glutamine. The nonadherent cellular fraction was removed, and the remaining adherent CD45^+^ myeloid cells were treated with a 10-fold dilution of CSD peptide LTI-2355 (0.1 μM, 1.0 μM, and 10.0 μM), which is a 15-mer capped with two D-alanines with an acetate counterion containing the sequence EGKASFTTFTVTKGS (Polypeptide, Torrance, CA, USA), 5 µg/mL UNO peptide (318897, NovoPro BioSciences, Shanghai, China), or the standard-of-care drug nintedanib (Ofev^®^, Boehringer Ingelheim, Ingelheim am Rein, Germany) at a clinically relevant dose of 80 nM.

Efferocytosis, phagocytosis, and proliferation assays. For the quantification of efferocytosis in cultured CD45^+^ myeloid cells from healthy donors, IPF, and PASC-F, cellular debris (defined as cellular material (≤225 μm^2^)) was labeled and quantified using IncuCyte 2021 software in an IncuCyte S3 system. Thirty (30)-minute pre-incubation with 10 µM cytochalasin D (Sigma-Aldrich, St. Louis, MO, USA, C8273-1MG) was used as a negative control. For the quantification of phagocytosis, CD45^+^ myeloid cells from healthy donors, IPF, and PASC-F were cultured with 0.01 mg/mL pHrodo Red *Staphylococcus aureus* (SA), *E. coli*, or zymosan bioparticles (4619/4615/4617, Sartorius, Bohemia, NY, USA); the total red object integrated intensity (red calibrated unit (RCU) × µm^2^/image) was measured after compensating for background fluorescence at 1 h intervals using the IncuCyte S3 system (Essen BioScience, Ann Arbor, MI, USA). Proliferation/viability was quantified in cultured CD45^+^ myeloid cells from healthy donors, IPF, and PASC-F with IncuCyte^®^ NucLight Rapid Red Dye (4717, Sartorius, Göttingen, Germany). LTI-2355, UNO, nintedanib, or the appropriate control substance was added at the indicated concentration at the start of these assays, unless otherwise stated.

Proteomic analysis. Cell-free tissue culture supernatants were collected after 3 days of culture. Pro-inflammatory and pro-fibrotic mediators were measured in 50 µL of cell-free tissue culture supernatants using a 37-Plex Bio-Plex Pro™ Human Inflammation Panel 1 #171AL001M (Bio-Rad Laboratories, Hercules, CA, USA) according to the manufacturer’s instructions. Briefly, the supernatant was incubated for 1 h at 24 °C with capture antibodies that were covalently coupled to magnetic beads. The unbound supernatant was washed before sequential incubation with a biotinylated detection antibody and a streptavidin–phycoerythrin conjugate. Data were acquired using a Bio-Plex 200 reader (Bio-Rad Laboratories, Hercules, CA, USA). LTI-2355, UNO, nintedanib, or the appropriate control substance was added at the indicated concentration at the start of these assays, unless otherwise stated.

## 3. Results

**Figure 1:**
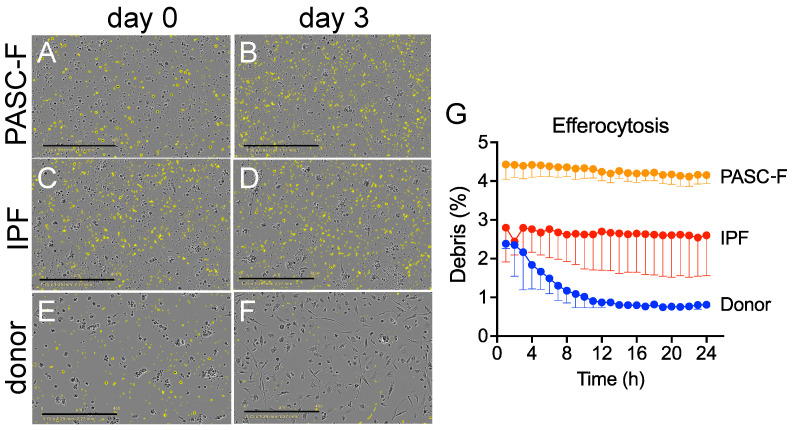
Impaired removal of dead cells and cellular debris by cultured IPF and PASC-F CD45^+^ myeloid cells. CD45^+^ myeloid cells were enriched from healthy donor lungs and IPF and PASC-F lung explants and cultured for 3 days, with live imaging conducted every hour. Dead cells and cellular debris, defined as particulate material ≤ 225 μm^2^, were labeled and quantified using IncuCyte 2021 software. Representative images of dead cells and cellular debris (colored yellow) in cultures of PASC-F myeloid cells at days 0 (**A**) and 3 (**B**); cultured IPF myeloid cells at days 0 (**C**) and 3 (**D**); and healthy donor myeloid cells at days 0 (**E**) and 3 (**F**) of culture. Quantification of debris clearance by healthy donor, IPF, and PASC-F myeloid cells during 24 h of culture is summarized in panel (**G**). Data are presented as the medians, with interquartile ranges; statistical significance was determined using ANOVA and Kruskal–Wallis tests.

The aberrant activity and altered morphology of CD45^+^ lung myeloid cells from IPF and PASC-F patients were compared with those of healthy donor CD45^+^ myeloid cells. Compared with healthy donor myeloid cells (Figure 1A,B) at days 0 and 3 in culture, IPF (Figure 1C,D) and PASC-F (Figure 1E,F) myeloid cells exhibited decreased motility and adherence, as well as altered cell morphology. To examine the intrinsic myeloid cell activity, the ability of myeloid cells to clear dead cells and cellular debris (i.e., particulate matter ≤ 225 μm^2^) was quantified with live imaging during the first 24 h of culture (Figure 1A–F). The area under the curve analysis showed impaired clearing of dead cells and particulate debris by IPF myeloid cells when compared with healthy donor myeloid cells (*p* < 0.001, Figure 1G).

**Figure 2:**
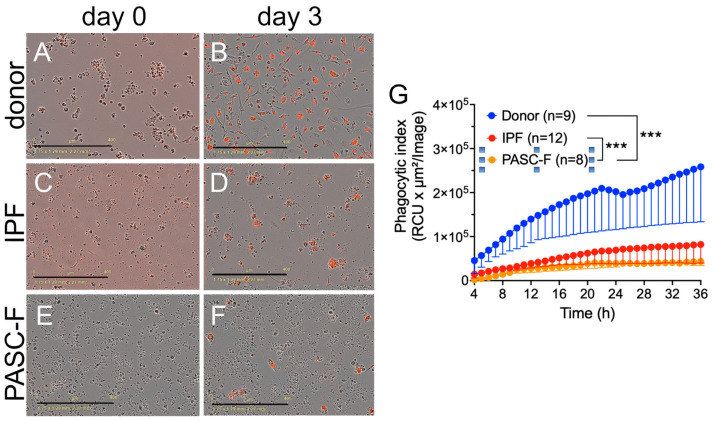
Impaired phagocytosis of bacterial antigen-coated bioparticles by IPF and PASC-F myeloid cells. CD45^+^ myeloid cells were enriched from healthy donor lungs and IPF and PASC-F lung explants and cultured for 3 days, with imaging conducted every hour in the presence of pHrodo SA beads. The uptake of bioparticles was quantified by measuring the red image fluorescent signal using IncuCyte 2021 software. Representative images of bioparticle uptake (red label signal) in cultures of PASC-F myeloid cells at days 0 (**A**) and 3 (**B**); cultured IPF myeloid cells at days 0 (**C**) and 3 (**D**); and healthy donor myeloid cells at days 0 (**E**) and 3 (**F**) of culture. Quantification of the pHrodo emission by healthy donor, IPF, and PASC-F myeloid cells during 24 h of culture (**G**). Data are presented as the means ± SEM of three replicates; statistical significance was determined using ANOVA and Kruskal–Wallis tests; *** *p* < 0.001.

**Figure 3:**
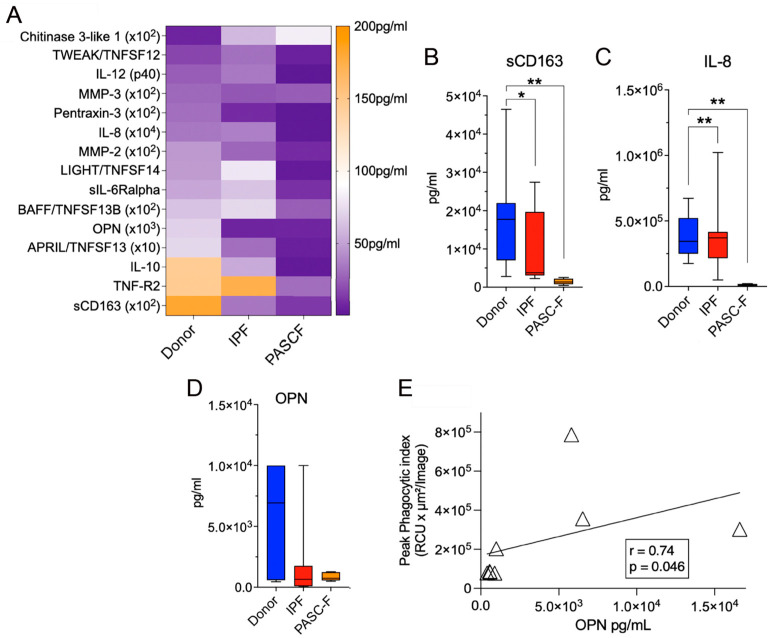
Soluble mediators generated by healthy donor, IPF, and PASC-F myeloid cells. CD45^+^ myeloid cells enriched from healthy donor, IPF, and PASC-F lung samples or explants were allowed to adhere in tissue culture plates for 24 h. The nonadherent cellular fraction was removed, and the adherent myeloid cells were maintained in culture for an additional 5 days. Protein levels were measured using Bio-Plex (Bio-Rad) in cell-free culture supernatants (**A**). Statistically significant differences in the levels of sCD163 (**B**), IL-8 (**C**), and osteopontin (OPN) (**D**) were observed between healthy donor and fibrotic lung myeloid cells. The correlation of the OPN levels and the peak phagocytic activity by IPF myeloid cells at 48 h of culture is shown in (**E**). Statistical significance was determined using ANOVA and Mann–Whitney U tests (**B**–**D**) and Spearman’s correlation (**E**); * *p* < 0.05, ** *p* < 0.01.

Impaired phagocytic activity of CD45^+^ myeloid cells enriched from IPF and PASC-F lung explants. To determine whether impaired debris cleaning was due to an impaired phagocytic response, we added pHrodo-labeled *Staphylococcus aureus* (SA) beads to isolated myeloid cells. In the presence of pHrodo SA bioparticles, healthy donor myeloid cells and IPF-derived myeloid cells showed a time-dependent increase in the phagocytic index (Figure 2A (day 0 in culture), Figure 2B (day 3 in culture), and Figure 2G). In contrast, IPF lung myeloid cells exhibited delayed and impaired intrinsic phagocytic activity in culture (*p* < 0.001; Figure 2C (day 0), Figure 2D (day 3), and Figure 2G). Impaired phagocytic activity was also observed in PASC-F primary myeloid cells (Figure 2E (day 0), Figure 2F (day 3), and Figure 2G). To determine whether the uptake of foreign particles was related to an overall impairment of myeloid cells or whether it was specific to SA or the toll-like receptor (TLR)-2/4 mechanism, we repeated experiments with *E. coli*- and zymosan-coated bioparticles. Coated bioparticle uptake by myeloid cells showed a patient-specific but not an agonist-coated bead-specific response, indicating an overall intrinsic defect in the function of IPF and PASC-F myeloid cells (Supplementary Figure S1). Unlike healthy donor myeloid cells in culture, IPF myeloid cells formed aggregates with dead cells and debris, appearing to move around the culture plate with this cellular debris attached to the cell surface (Supplementary Figure S2). Decreased synthetic activity in IPF and PASC-F myeloid cells was observed following the analysis of several soluble pro-inflammatory and pro-fibrotic mediators, as shown in Figure 3A. The one exception among the mediators analyzed was the pro-fibrotic factor chitinase 3-like 1, which was elevated in cultures of both IPF and PASC-F myeloid cells compared with healthy donor myeloid cells (Figure 3A). The total soluble CD163 (sCD163) and IL-8 levels in cultures of IPF and PASC-F myeloid cells were statistically significantly lower when compared with healthy donor myeloid cells (Figure 3B,C). While secreted osteopontin (OPN) levels were lower in cultures of IPF and PASC-F myeloid cells (Figure 3D), the levels of this cytokine were positively correlated with the phagocytic activity of myeloid cells from healthy donor and fibrotic lung samples (Figure 3E; *p* = 0.046).

**Figure 4:**
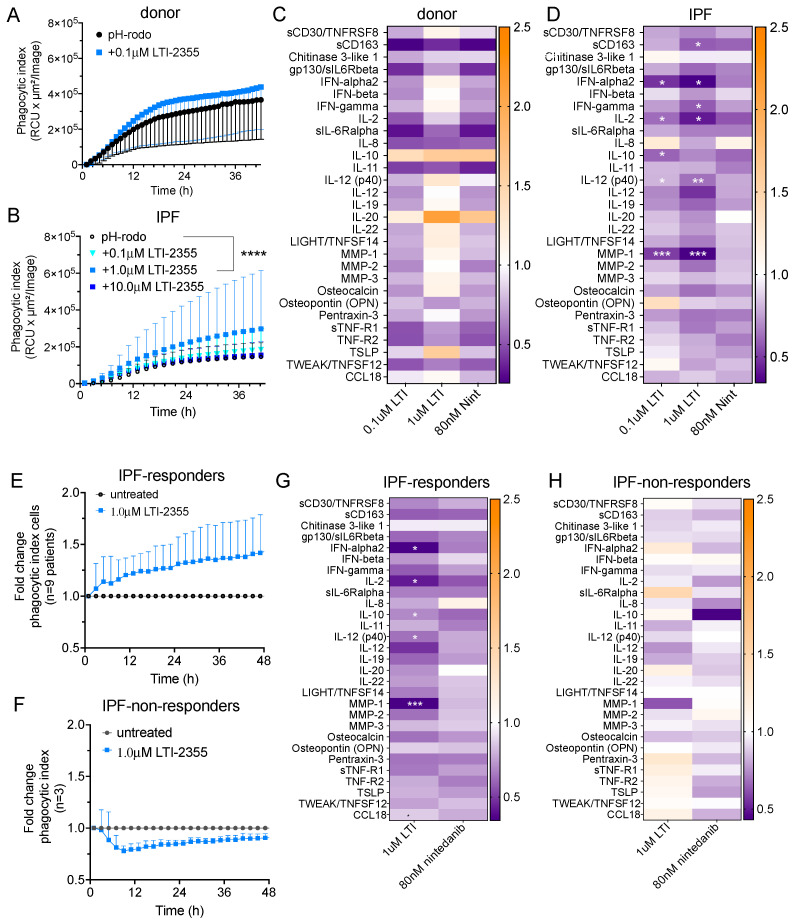
LTI-2355 significantly enhanced the phagocytic activity and decreased the soluble mediator levels in enriched CD45+ myeloid cells from IPF patient explants. CD45^+^ myeloid cells were enriched from healthy donor lung (**A**) and IPF (**B**) explant tissue and treated with 0.1–10.0 μM LTI-2355 for 48 h. To quantify the phagocytic activity, enriched myeloid cells were cultured in the presence of pHrodo SA beads and imaged every hour in an IncuCyte S3 system. To determine the effect of LTI-2355 and nintedanib on the generation of inflammatory and pro-fibrotic mediators, cell-free culture supernatants were collected from cultured myeloid cells enriched from healthy donor (**C**) and IPF lung explant tissue (**D**) and were analyzed via Bio-plex (Bio-Rad). During these experiments, IPF patient myeloid cells from three patient lung explants did not appear to respond to the single LTI-2355 treatment. Consequently, IPF myeloid cells were separated into responders (**E**,**G**) and non-responders (**F**,**H**), and the phagocytic index changes (normalized to the pHrodo control (fold-change phagocytic index)) and synthetic activity are shown. The statistical significance of the phagocytic index changes was determined using ANOVA and Kruskal–Wallis tests; **** *p* < 0.0001. Protein expression was measured via Bio-Plex (Bio-Rad) and presented as the median fold change compared with the untreated controls. Two-way ANOVA with Bonferroni correction for multiple comparisons compared with the control or LTI-2355 (blue); * *p* < 0.05, ** *p* < 0.01, *** *p* < 0.001.

LTI-2355 increased the phagocytic activity and reduced the levels of pro-inflammatory and pro-fibrotic mediators secreted by lung myeloid cells from IPF patients. Healthy donor myeloid cells exhibited a time-dependent increase in phagocytic index, which was not enhanced by the presence of LTI-2355 at a dose of 0.1 μM (Figure 4A). In contrast, LTI-2355 significantly enhanced the phagocytic activity of IPF myeloid cells at a dose of 1.0 μM, but not at lower (i.e., 0.1 μM) or higher (i.e., 10 μM) doses (Figure 4B). Since the increased phagocytic activity of lung myeloid cells is also known to enhance the generation of pro-inflammatory and pro-fibrotic mediators, we examined the synthetic activity of healthy donor and IPF myeloid cells. An analysis of supernatants from healthy donor (Figure 4C) and IPF (Figure 4D) myeloid cells showed an overall statistically significant reduction in the presence of several mediators generated by IPF myeloid cells, but not by healthy donor myeloid cells. Specifically, sCD163, IFN-α2, IFN-γ, IL-2, IL-10, IL-12p40, and MMP-1 levels were significantly lower in the LTI-2355-treated cultures of IPF myeloid cells compared with the control untreated IPF myeloid cells (Figure 4D). Interestingly, nintedanib (at 80 nM) did not significantly reduce the levels of any of the soluble mediators measured in the cultures of treated IPF myeloid cells compared with untreated IPF myeloid cells (Figure 4D). It was also interesting to note that there were IPF myeloid cells that did not appear to respond to the presence of a single LTI-2355 treatment after 48 h in culture [Figure 4E (responders) versus Figure 4F (non-responders)]. An analysis of the soluble pro-inflammatory mediators in these two groups of IPF patients confirmed that LTI-2355 significantly inhibited the generation of pro-inflammatory and pro-fibrotic mediators in the responder group, but not in the non-responder group (Figure 4G and Figure 4H, respectively). However, the addition of LTI-2355 every 24 h in culture for the duration of the analysis was found to significantly increase the phagocytic activity of these IPF non-responders (Supplementary Figure S3), highlighting the potential of the repeated administration of LTI-2355 to IPF myeloid cells as a strategy to enhance the phagocytic properties of these cells.

**Figure 5:**
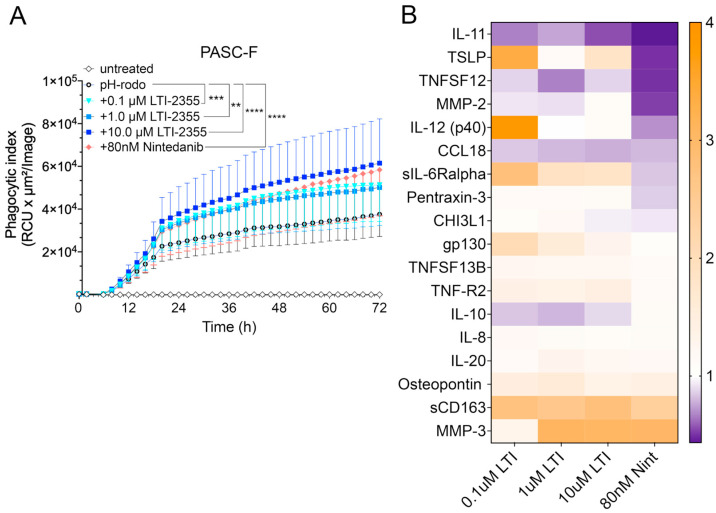
LTI-2355 increased the phagocytic activity of PASC-F myeloid cells. CD45^+^ myeloid cells were enriched from PASC-F patients and treated with 0.1 μM, 1 μM, and 10 μM LTI-2355 or nintedanib (80 nM) in culture for 3 days, with imaging conducted every hour in the presence of pHrodo SA beads. The uptake of bioparticles was quantified by measuring the red image fluorescent signal using IncuCyte 2021 software. Quantification of the pHrodo emission by PASC-F myeloid cells in each treatment group (**A**). The pro-inflammatory protein ratio compared with the untreated controls was quantified via Bio-plex in the cell culture supernatants of stimulated myeloid cells (**B**) (n = 5). Statistical analysis was determined using ANOVA and Kruskal–Wallis tests compared with the baseline phagocytic activity (**A**); ** *p* < 0.01, *** *p* < 0.001, **** *p* < 0.001 compared with the pHrodo control group.

LTI-2355 enhanced the phagocytic properties and modulated the synthetic properties of PASC-F myeloid cells. The addition of LTI-2355 to cultured PASC-F myeloid cells dose-dependently increased the phagocytic activity of these cells; the maximum PASC-F myeloid cell phagocytic activity was observed at 10 μM of LTI-2355 (Figure 5A). Unlike IPF myeloid cells, the presence of LTI-2355 had a more modest effect on the synthetic activity of pro-inflammatory and pro-fibrotic mediators generated by cultured PASC-F myeloid cells (Figure 5B). The data were not statistically significant. Together, these data indicated that LTI-2355 significantly altered the phagocytic activity and more modestly altered the synthetic activity of PASC-F myeloid cells.

**Figure 6:**
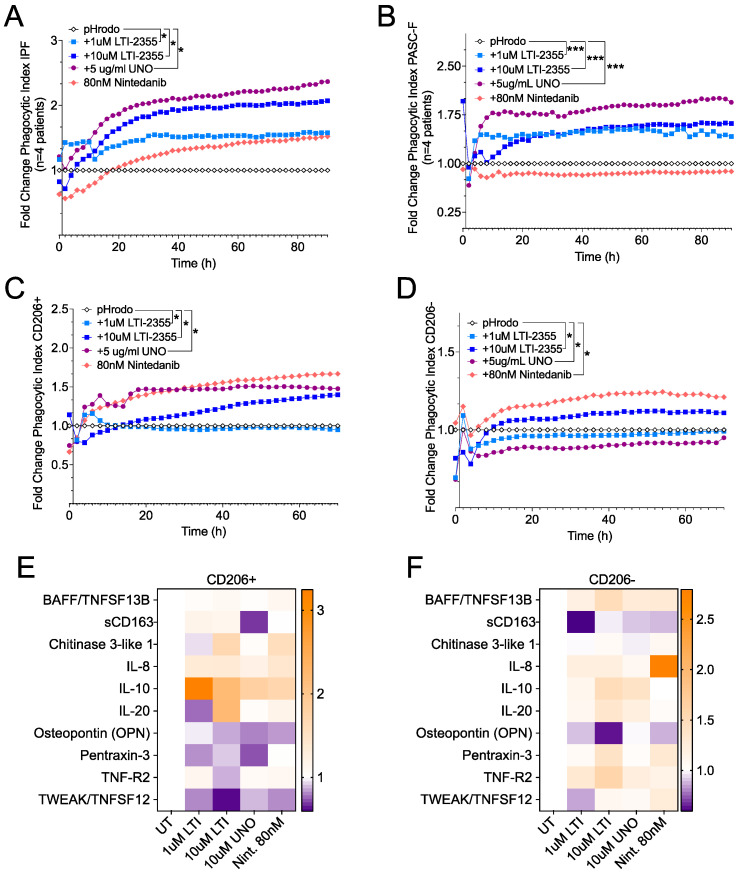
Role of CD206 in the response of IPF and PASC-F myeloid cells to LTI-2355. CD45^+^ myeloid cells were enriched from IPF and PASC-F lung explant tissue and cultured for 3 days, with imaging conducted every hour in the presence of pHrodo SA beads. The uptake of bioparticles was quantified by measuring the red image fluorescent signal using IncuCyte 2021 software. Quantification of the pHrodo emission by IPF (**A**) and PASC-F (**B**) myeloid cells during 92 h of culture was normalized to the pHrodo control (fold change phagocytic index). Fold change in the phagocytic index of pHrodo emission by CD206-positive (CD206^+^) (**C**) and CD206-negative (CD206^−^) (**D**) IPF and PASC-F CD45^+^ enriched myeloid cells normalized to the pHrodo control. Pro-inflammatory and pro-fibrotic mediator ratios in cell-free tissue culture supernatants from the LTI-2355-, UNO-, and nintedanib-treated (80 nM) CD206^+^ (**E**) and CD206^−^ (**F**) myeloid cells compared with the untreated control groups of both myeloid cell types. Data are presented as the means (with the standard error of the mean). Multiple comparisons with Dunn’s correction; * *p* < 0.05, *** *p* < 0.001 all conditions vs. pHrodo control (untreated—UT).

Role of CD206 in LTI-2355-mediated effects on IPF and PASC-F myeloid cells. To explore whether CD206 was required for the effects of LTI-2355 on the lung myeloid cell phagocytic and synthetic function, we compared the effects of LTI-2355 with those of UNO, which is a CD206 (MRC1)-binding peptide. As shown in Figure 6, both LTI-2355 and UNO significantly enhanced the phagocytic activity of IPF myeloid cells (Figure 6A) and PASC-F myeloid cells (Figure 6B). In cultures composed of CD206-positive myeloid cells, both LTI-2355 and UNO significantly enhanced the phagocytic activity of these myeloid cells (Figure 6C). In cultures composed of CD206-negative myeloid cells (from either IPF or PASC-F), the presence of LTI-2355 or nintedanib, but not of UNO, significantly increased the phagocytic activity of this fraction of myeloid cells (Figure 6D). Interestingly, nintedanib enhanced the phagocytic activity of CD206-negative but not CD206-positive lung myeloid cells. Further, UNO suppressed the phagocytic activity of CD206-negative lung myeloid cells compared with the pHrodo control group (Figure 6D). LTI-2355 and UNO inhibited the synthetic activity of CD206-positive lung myeloid cells, as shown in Figure 6E; however, only LTI-2355 showed inhibitory effects on the generation of sCD163 and OPN in CD206-negative lung myeloid cells (Figure 6F). Finally, we observed that neither peptide nor nintedanib altered the proliferation of lung myeloid cells from IPF and PASC-F lung explants over 48 h in culture (Supplementary Figure S4). Overall, these data indicate a broad mechanism of action of LTI-2355; however, the presence of CD206 on myeloid cells appears to be required for its effects on both the phagocytic and synthetic properties of these cells.

## 4. Discussion

In both PASC-F and IPF, persistent myeloid cell activation is a key driver of disease pathology, and RNAseq studies have demonstrated shared expression profiles of myeloid cells derived from these diseases [3,18,19]. IPF is a chronic, progressive lung disease characterized by excessive fibrosis and scarring of lung tissue, leading to respiratory failure. Currently, two standard-of-care therapies slow the rate of disease progression in IPF [20], and many other therapies targeting a variety of mechanisms are in development, including a CSD peptide, LTI-03 [21]. At the time of submission, the NIH research initiative RECOVER-TLC is underway in pursuit of defining PASC-F pathogenesis and facilitating the exploration of novel therapeutic interventions, some of which target inflammatory processes [22].

Unlike classic inflammatory lung diseases such as asthma, IPF does not exhibit robust inflammation; however, perturbed gene expression and aberrant activity of myeloid cells are observed [23]. While fibroblast activation and epithelial cell dysfunction are central to IPF pathogenesis [24], the role of the immune system and its contribution to disease initiation and progression remain under investigation, as evidence suggests that macrophages play a role in tissue remodeling and fibrosis. In the context of PASC-F, persistent activation of monocytes and macrophages has been observed, which likely contributes to chronic inflammation and metabolic dysfunction, driving fibrotic progression. These cells exhibit heightened inflammatory cytokine production (e.g., IL-6, TNF-α) and impaired phagocytosis, leading to prolonged immune activation [25] Furthermore, there is evidence that aging-associated immune dysregulation including senescence and chronic low-grade inflammation may contribute to the persistence of fibrosis in IPF [26].

While this is the first study to explore the effects of CSD peptides on the phagocytic and synthetic activity of myeloid cells derived from IPF and PASC-F human lung tissue, CSD peptides have been found to suppress enhanced migration of monocytes in mouse dermal and lung fibrosis models [27]. In IPF, macrophages contribute to aberrant tissue remodeling and fibrosis, while in PASC-F, sustained monocyte activation fuels chronic inflammation and metabolic dysfunction. Both conditions exhibit myeloid-driven dysregulation of wound healing, extracellular matrix deposition, and pro-inflammatory signaling, with aging-associated immune dysfunction likely exacerbating disease progression.

Herein, we demonstrate that CD45^+^ myeloid cells isolated from IPF and PASC-F lung explant tissue (sourced from random cores of diseased lung tissue biopsied from discarded IPF or PASC-F lungs following transplantation and banked for IRB-approved research use) have an impaired capacity to clear autologous cellular debris and phagocytose foreign bioparticles in in vitro assays. While healthy donor myeloid cells efficiently phagocytosed cellular debris and pathogen-coated bioparticles, IPF and PASC-F myeloid cells appeared to bind but not engulf cellular debris in the culture system, resulting in the clumping of this material on the surface of these cells. IPF and PASC-F myeloid cells were also observed traversing around the culture system with this cellular material attached to their surface. The uptake of pHrodo bioparticles was also impaired independently of the nature of the bioparticle, highlighting a myeloid cell-intrinsic functional impairment. LTI-2355 dose-dependently improved the efferocytotic and phagocytic properties of both IPF and PASC-F lung myeloid cells, while this modified CSD peptide concomitantly reduced the release of pro-inflammatory and pro-fibrotic mediators by these cells. This dual effect of LTI-2355 might be therapeutically significant in the modulation of fibrotic processes in both IPF and PASC-F.

Our results in relation to clinical fibrotic lung disease confirm findings in mice [28], in which the innate immune response is blunted in bleomycin-exposed mice. Moreover, macrophages loaded with liposomes containing dexamethasone attenuated bleomycin-induced pulmonary fibrosis in mice by reducing the activation of pro-fibrotic macrophages [29,30]. Mechanistically, exposure to a high burden of apoptotic cells in IPF and PASC-F might have altered the activation state of lung myeloid cells via the downregulation of efferocytosis receptors such as CD11b, thereby reducing phagocytic capacity [31,32]. However, in the present study, we examined the overall adherent lung myeloid population isolated from human lung explants without distinguishing between the satiated (i.e., CD11b^low^ phenotype) and activated (i.e., CD11b^high^ phenotype) phenotypes. Schloesser et al. [33] showed that a suppressed macrophage phenotype was mediated through a cell contact-dependent interaction with senescent fibroblasts. While our results indicate that primary IPF and PASC fibrosis myeloid cells showed an intrinsic suppression of myeloid cell activity, future studies will address the role of the senescent environment on the impairment of IPF and PASC-F lung myeloid cells and whether LTI-2355 alters the functionality of these myeloid cells so that these cells exhibit a pro-resolving phenotype [34].

Studies in murine models have delineated macrophages into M1 and M2 macrophages, respectively, activated by pathogens and Th2 cytokines [35]. While M1 macrophages contribute to inflammation by secreting tumor necrosis factor (TNF)-α, interleukin (IL)-1β, IL-6, inducible NO synthase (iNOS), and matrix metalloproteinases (MMPs), M2 macrophages release anti-inflammatory or pro-resolving mediators, including IL-10, transforming growth factor (TGF)-β, arginase (ARG)-1, and mannose receptor (CD206), thereby mediating wound repair, tissue remodeling, and fibrosis [36,37]. CD206, or MRC1, consists of a fibronectin type II domain (FNII) that interacts with collagen, a cysteine-rich domain that binds sulfated proteoglycans, and a lectin domain composed of eight carbohydrate recognition domains that bind mannose. The importance of the CD206 receptor has been shown in peptide studies. Both Scodeller et al. and Jaynes et al. demonstrated that small peptides, UNO, and riptide-182, respectively, are taken up in a CD206-dependent manner into the cell, and M2-like macrophages are reprogrammed into an M1-like macrophage phenotype [38,39]. The downregulation of cytokine production by LTI-2355 is consistent with a study by Takamura et al., who demonstrated that the downregulation of Cav-1 correlated with increased cytokine levels, which are reversible via Cav-1 transgene administration [40]. However, we are cognizant that pro-fibrotic macrophages in vivo are not completely identical cell populations compared with M2-like macrophages in vitro. Additionally, culture conditions affect macrophage polarity, which is why we chose to perform our experiments on the whole myeloid cell population instead of focusing on M2 macrophages [20]. Furthermore, in vitro cultures of macrophages lack alveolar epithelial cells, which has been shown to decrease AM phagocytosis and cytokine production in a TGF-β-dependent manner via cell-to-cell contact; the indirect production of surfactant proteins can initiate the phagocytosis of apoptotic cells and pathogens [41,42].

In a further study, we observed that LTI-2355 had superior effects on the phagocytic activity of myeloid cells compared with nintedanib. Moreover, supernatants from healthy donor (Figure 4C) and IPF (Figure 4D) myeloid cells showed an overall statistically significant reduction in the sCD163, IFN-α2, IFN-γ, IL-2, IL-10, IL-12p40, and MMP-1 production by IPF myeloid cells, but not by healthy donor myeloid cells. A trend toward suppression of these factors by LTI-2355 was noted in PASC-F myeloid cells. We hypothesize that the lack of response by PASC-F myeloid cells as compared with IPF myeloid cells may be due to stronger proteolytic activity, as indicated by higher baseline MMP-3 levels, which may be destructive to the peptide itself.

In mice, nintedanib has been described to prevent macrophage activation and differentiation toward an M2 phenotype (presumably via the blockade of CSF1R), thereby indirectly preventing fibroblast activation without affecting M1 markers [43,44,45,46]. The relevance of peptide-based macrophage targeting was confirmed by Ghebremedhin et al., who demonstrated that a mannose receptor-targeting peptide RP-832c inhibited M2 macrophage activation and attenuated fibrosis in a bleomycin model of pulmonary fibrosis [47]. As new treatment options emerge in IPF, directed at inducing apoptosis in senescent lung cells via a senolytic mechanism [48], it is becoming clear that the restoration of efferocytosis and phagocytosis in lung myeloid cells is needed to remove apoptotic cells from the lung. However, finding the exact mechanisms needed to modulate the myeloid cell activity in this manner requires further investigation in IPF, PASC-F, and other progressive ILDs. Thus, future studies addressing the interplay between myeloid cells, alveolar type 2 epithelial cells, basaloid cells, and (myo-)fibroblasts via the establishment of a microenvironment favoring homeostasis are essential in the search for new treatments for IPF.

One limitation of our study is that the myeloid cells were obtained from end-stage IPF and PASC-F explants, which precludes our analysis of inflammatory/immune cells in earlier phases of the disease. In addition, the IPF patients were older than the healthy donors, which might influence the phagocytic and synthetic properties of myeloid cell populations. While a single treatment with LTI-2355 significantly increased phagocytosis in most myeloid cell preparations from IPF and PASC-F patients compared with the untreated conditions, LTI-2355 did not restore the functional activity levels of IPF and PASC-F myeloid cells to those observed in healthy donor myeloid cells. An increased fluorescent signal after LTI-2355 treatment might indicate an increased capacity of active phagocytes to engulf bioparticles or a phenotype switch of the phagocytes in culture toward an active phenotype, but the current studies have not confirmed whether either or both mechanisms are activated by LTI-2355 treatment. Finally, we did not examine the response of subpopulations of macrophages identified by several groups [3,4,5,49]. This limitation was a consequence of our inability to reproducibly isolate a sufficient amount of viable Mertk^+^, TREM2^+^, and other distinct macrophage subtypes for the functional studies described herein.

In conclusion, we demonstrated that the CSD peptide LTI-2355 enhances the phagocytic activity and modulates the synthetic activity of IPF and PASC-F myeloid cells in a CD206-dependent manner, indicating a novel mechanism of action, by which CSD peptides may confer therapeutic benefits. Combined with the previously reported therapeutic effects of CSD on the inhibition of fibroblast activation and the restoration of alveolar epithelial cells, these data indicate the potential of CSDs to modulate myeloid cell activity in IPF and PASC-F. Alternative approaches to enhance/restore anti-fibrotic macrophage function should focus on restoring phagocytic activity. The disruption of CD47 receptor and SIRPα agonist interaction to enhance macrophage functions (including phagocytosis, antigen presentation, and ADCC) has shown promise in scleroderma [50]. While macrophages or myeloid cells have been the target in IPF, including Galecto’s galectin-3 inhibitor GB0139 and Hoffmann-La Roche’s zinpentraxin (RPM-151; a recombinant human pentraxin-2), neither of these targeting strategies have affected the primary endpoint of slowing the forced vital capacity decline in clinical trials. Since adverse effects were observed in the treatment arms of these trials, more research is required to understand the overall role of macrophage/myeloid cell biology in IPF patients and other progressive ILDs. In summary, LTI-2355 enhances primary lung myeloid cell functional activity and modulates the pro-inflammatory and pro-fibrotic properties of these immune cells in IPF and PASC-F. Additional research is needed to determine whether targeting myeloid cell activity could slow fibrotic lung disease progression and thereby provide therapeutic benefit.

## Figures and Tables

**Table 1:** Patient characteristics.

	Controls	IPF	PASC-F
Age (mean, (SD))	51.6 (14.5)	66.0 (15.8)	56.5 (16.0)
Gender (F/M (% male))	0/7 (100%)	2/7 (77.8%)	1/7 (85.7%)
Smoking history (S/F/NS)	1/1/5	0/2/7	0/1/7

S—smoker; F—former smoker; NS—never smoker.

## Data Availability

Data can be made available by contacting the corresponding author.

## Supplementary Materials

The following supporting information can be downloaded at: https://www.mdpi.com/article/10.3390/biomedicines13040796/s1, Figure S1: Impaired phagocytosis of bioparticles by IPF myeloid cells was independent of the fungal or bacterial ligands coating the pHrodo beads; Figure S2: Illustration of aggregate formation and dragging phenotype by IPF myeloid cells; Figure S3: Effect of repeated LTI-2355 administration on the phagocytic index of IPF CD45^+^ myeloid cells. Figure S4: In vitro proliferation of IPF myeloid cells was not observed in these cultured cells following exposure to LTI-2355, UNO, or nintedanib.

## Abbreviations

AM	Alveolar macrophage
CAV	Caveolin
CD	Cluster of differentiation
CH3IL1	Chitinase 3-like-1
COPD	Chronic obstructive pulmonary disease
CSD	Caveolin scaffolding domain
DMEM	Dulbecco’s modified Eagle’s medium
ECM	Extracellular matrix
FABP	Fatty acid-binding protein
IL	Interleukin
IPF	Idiopathic pulmonary fibrosis
MERTK	MER proto-oncogene tyrosine kinase
MMR/MRC	Mannose receptor
MMP	Matrix metalloprotease
Mo-MA	Monocyte-derived macrophage
PASC-F	Post-acute sequelae of COVID fibrosis
RCU	Red calibrated unit
SA	*Staphylococcus aureus*
sCD	Soluble CD
SPP/OPN	Osteopontin
TLR	Toll-like receptor
TNF	Tumor necrosis factor
TR-AM	Tissue-resident alveolar macrophage
