# Supplementary material for: Caveolin Scaffolding Domain (CSD) Peptide LTI-2355 Modulates the Phagocytic and Synthetic Activity of Lung-Derived Myeloid Cells in Idiopathic Pulmonary Fibrosis (IPF) and Post-Acute Sequelae of COVID Fibrosis (PASC-F)
Source: Biomedicines. 2025 Mar 26;13(4):796. doi: 10.3390/biomedicines13040796 (PMC12024842; doi:10.3390/biomedicines13040796)
Supplement: Supplementary file 1 [file biomedicines-13-00796-s001.zip › biomedicines-3518468-supplementary.docx]

Supplementary Material


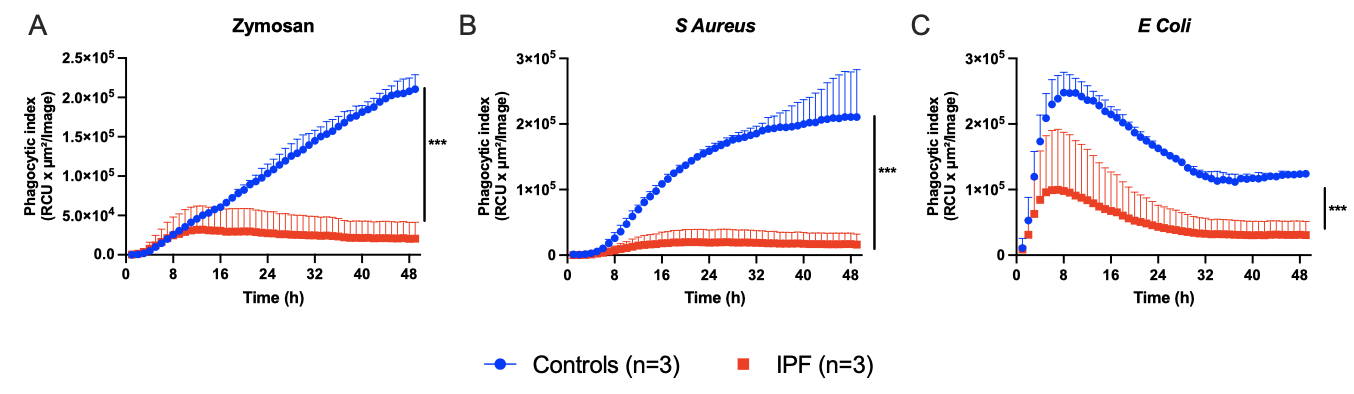
**Figure S1.** Impaired phagocytosis of bioparticles by IPF myeloid cells was independent of the fungal or bacterial ligands coating the pH-rodo beads. Myeloid cells from IPF lung explant tissue were enriched and cultured for 2 days with imaging being conducted every hour in the presence of pH-rodo zymosan (**A**), *S Aureus* (**B**), or *E Coli* (**C**) beads. The uptake of bioparticles was quantified by measuring red image fluorescent signals using IncuCyte 2021 Software. The quantification of pH-rodo emission by control (n = 3) and IPF (n = 3) myeloid cells during 48h of culture (**A**). Data are presented as medians of three replicates with IQR. Mann–Whitney U testing; **** *p* < 0.0001.


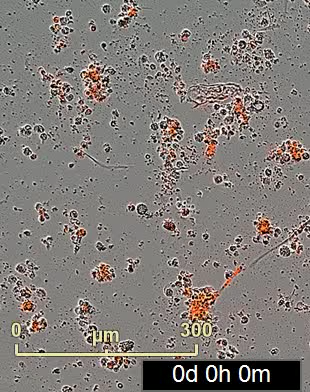
.

**Figure S2.** Illustration of aggregate formation and dragging phenotype by IPF myeloid cells. Immune cells from lung explant tissue were enriched and cultured for 3 days. Imaging shown every 6 h in the presence of pH-rodo SA beads.


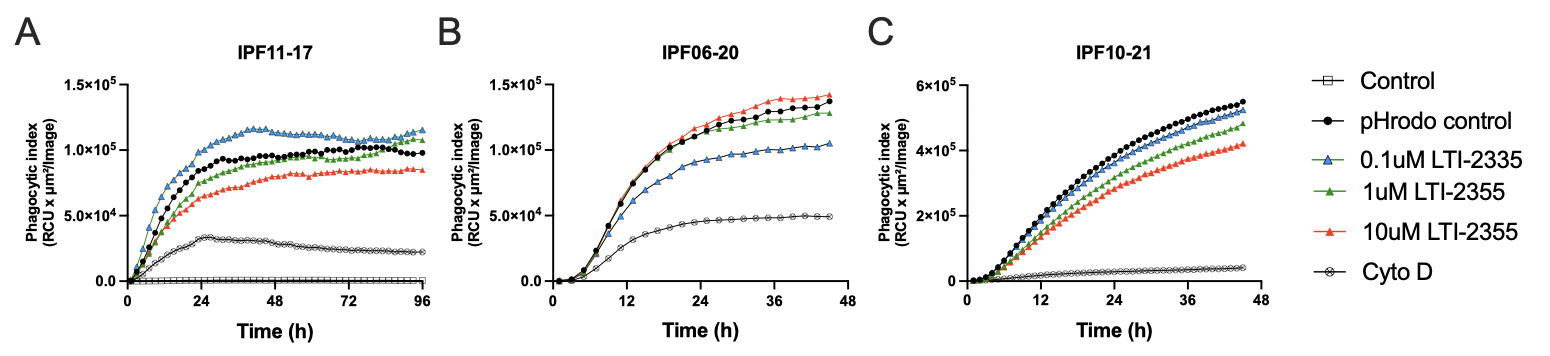
.

**Figure S3.** Effect of repeated LTI-2355 administration on the phagocytic index of IPF CD45^+^ myeloid cells. CD45^+^ myeloid cells were enriched from IPF lung explant tissue and were enriched and stimulated with either 0.1, 1, or 10 LTI-2355 μM at 24 h intervals during this experiment. CD45^+^ myeloid cells from these IPF patients were non-responsive to one administration of LTI-2355 into the cultured cells, but the repeated administration of this CSD peptide every 24 h enhanced the phagocytic activity of two out of the three patient myeloid lines examined. The phagocytic index was quantified by live cell imaging using IncuCyte 2021 Software and detected as the red image fluorescent signal resulting from the uptake of pH-rodo SA bioparticles.

.
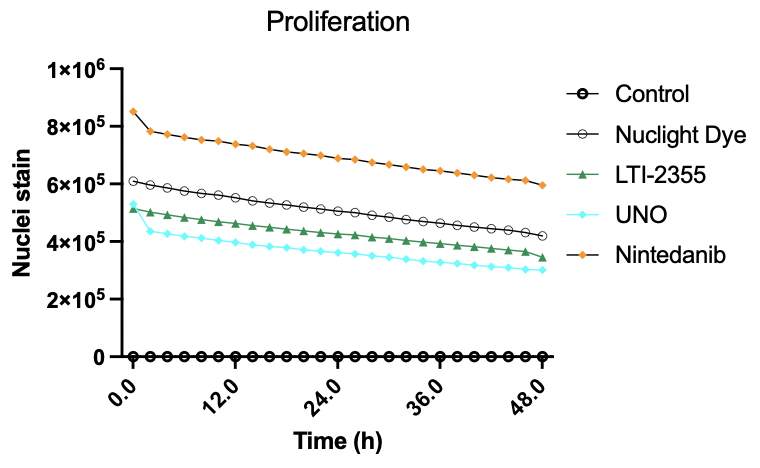


**Figure S4.** In vitro proliferation of IPF myeloid cells was not observed in these cultured cells following exposure to LTI-2355, UNO, or nintedanib. CD45^+^ myeloid cells enriched from IPF lung explant tissue (n = 4) were enriched and stimulated after 24h in culture with LTI-2355, nintedanib, or UNO; responses were compared to control medium. Proliferation was quantified with IncuCyte^®^ NucLight Rapid Red Dye (IncuCyte) for 48 h with imaging every two hours. Proliferation was quantified by measuring Red Calibrated Unit (RCU) based on the red image fluorescent signal cell-by-cell analysis using IncuCyte 2021 Software.
